# Society Reports

**Published:** 1872-05-15

**Authors:** 


### Society Reports.

American Medical Association. — The following items, gleaned from the proceedings of the recent meeting of this great national organization, will be found of general interest to our readers:

Shortly after 11 o'clock a.m., the meeting was called to order by the President, Dr. D. W. Yandell, of Kentucky.

The Right Rev. William Bacon Stevens, M.D., D.D., D.C.L., Bishop of the Protestant Episcopal Church in Pennsylvania, then opened the proceedings with prayer.

The address of welcome on behalf of the medical profession of Philadelphia, was delivered by Professor R. E. Rogers, M.D., Chairman of the Committee of Reception, as follows:

Gentlemen — Delegates to the American Medical Association: It is with unfeigned pleasure that, as Chairman of the Committee of Reception, on behalf of the medical profession of Philadelphia, I extend to you a sincere and cordial welcome to our midst.

Gathered to-day in this hall are representatives of our profession from every quarter of this vast country, reaching from the city of the "Golden Gate" to the capes of the Chesapeake; from the granite hills of Maine to the waters of the Gulf of Mexico; covering a domain of twenty-four parallels of latitude and fifty degrees of longitude, and embracing a population of whose health its members are the guardians, numbering not less than thirty-eight millions of men, women, and children.

With these boundaries, this area possesses features of climate, a meteorology and a geology more diversified and extensive than those of any other single country on the face of the earth. What may we not hope of benefit to science and humanity by observations made upon these in their connection with disease and hygiene, by so many intelligent laborers in the field?

Meeting here, on this twenty-fifth anniversary of the organization of the American Medical Association, there are brought together men of every type, of high tone, loyal to their profession, strong men and true; men of sparkling genius, of varied and solid attainments — those who have grown gray in their vocation, laden with the golden harvest of a life's long experience, and those blessed with youth, energy, and ambition, engaged in laborious research, all ready to lay their individual contributions upon the altar of our profession.

In view of the reflections which here arise, what a spectacle of "moral grandeur" in possible achievements in the cause of suffering humanity does this assemblage present!

More than any other department of human knowledge does medicine appropriate the laws of the other positive sciences as parts of its organic whole, modifying and assimilating them, and finding in them sustenance, and warmth, and life. It is indeed the highest application of the truths of the other departments of knowledge, directed to the beneficent end of preserving health and assuaging physical pain. It may, perhaps, be not unaptly compared to some vast sea, receiving the contributions from a thousand perennial streams flowing from distant and widely separated regions, and then dispensing the commingled waters on the wings of the ever moving winds, to spread their vivifying, renovating showers over the wide circuit of the globe.

Since the organization of our Association, as by curious coincidence, a new era has been inaugurated; marvelous progress has been made in the developments of science bearing upon human comfort and the mitigation of disease. Thus, the electric telegraph, the vast extension of the iron track, the introduction of anæsthesia, and the device of various instruments of precision for the exploration and cure of the maladies of our race. These grand opportunities and grand advances have been within the reach of all. Therefore, as a natural result, our numbers have continued to increase, and the value of our transactions greatly to improve, thus securing an unabated prosperity to our organization.

Whatever qualifications may be made as to the amount of positive knowledge contributed through such annually recurring gatherings as these, it cannot be denied that they exercise personally a most wholesome influence upon the members of the profession. They serve to strengthen old friendships and form new ones, to dissipate prejudices, to sustain self-respect, and cultivate a spirit of charity and humility; and, through the interchange of opinions and the abrasion of thought, to brush away the cobwebs from the dusty corners of our brains; to sharpen the battle-axe which has grown rusty with disuse, and to lift us out of the monotonous groove of life in which we have been wont to move; leaving pleasant and cheering memories, which, like the Arctic twilight, lingers in the sky to meet the coming dawn of the next returning day.

The present is the third occasion of our meeting in the city of Philadelphia the earliest medical center of our country, which has long since become the vigorous and lusty sire of many a stalwart son. And here let us congratulate ourselves and the world at large upon the vindication of those principles which have ever guided her medical affairs. As the genuine coin is liable to counterfeit, so have unscrupulous men aimed to speculate upon her fair name by spurious imitations. Thanks to a wise legislation, the atmosphere has been purified, and the parties engaged in the fraudulent issue of diplomas have been deprived of their charters, and their institutions abolished.

The first meetings of this Association were marked by harmony and good will throughout; so may it be now; and may the blessing of the "Author of peace and Lover of concord" rest upon us and guide our deliberations.

Once more, then, my friends, offering you our warmest greetings, and a hearty welcome to our hospitalities, I give place to your further proceedings.

Dr. Edward Hartshorne, Chairman of the Committee of Arrangements, followed with some appropriate remarks, and asked the Secretary to read the list of those who had registered their names as members of the present meeting.

The President then read his annual address, concerning which we made some editorial comments in the previous issue of this journal.

Dr. H. F. Askew, of Delaware, then offered a resolution declaring that all questions of a personal character, including complaints and protests, and all questions concerning credentials, shall be referred at once to a Committee on Ethics, and without discussion, to be considered and reported on by such Committee.

The announcement of special reports and papers, and their reference to the appropriate sections, completed the work of the first general session.

At 3 o'clock p.m., the members assembled in the several Section rooms, and were busily engaged in the reading and discussion of reports and papers, embracing a great variety of subjects, the full value of which cannot be known until the publication of the Transactions.

In the evening a reception was given at Horticultural Hall by the Biological Society. In the foyer were arranged two long tables, on which were placed over one hundred powerful microscopes, containing specimens of pathology, anatomy, microscopic photography, etc.

Upon the platform at the end of this room were arranged five spectroscopes, in charge of Professor B. H. Rand, of the Jefferson Medical College, who exhibited the spectra of the different mineral and other substances, and of the electrical light.

---

SECOND DAY.

The Association was called to order at 10 o'clock a.m., the Vice-President, Dr. T. M. Logan, in the chair. The general session which followed was occupied chiefly in hearing the reports of the regular standing Committees, from which we gather the following items of general interest:

Dr. Casper Wister read the Treasurer's report. The expenditures during the year were $3,680; balance in hand, $1,005.

Dr. J. S. Weatherly, of Alabama, read the report of the Committee on Medical Education, which, after going briefly over the ground of the state of education in this country, and making a comparison with the European plans, threw out a number of suggestions that no more charters be granted to medical colleges which do not adopt the plan of medical education endorsed by the Association, and that applications be made to the Legislature for the revocation of the charters of such colleges as do not adopt the plan of the Association, and that no delegate be admitted into the conventions from an institution that has failed to adopt such plan. The report alluded to the rivalry between the various medical colleges, which induced them to lower the charges for education, and tended to make them throw abroad upon the world men who were totally unfit for the physician's duties.

Dr. Francis Gurney Smith, from the Committee on Prize Essay, announced that the successful essayist was Samuel R. Percy, M. D., who had struggled with the question "What Physiological Value has Phosphorus as an Organismal Element?"

Dr. T. Parvin, of Indiana, read the report of the Committee on Medical Literature, which asserted that while the medical profession of the country had a journalism which was honorable, worthy and able, yet that it was exposed to the danger of becoming local, provincial, was subjected to the influences of certain colleges, and that it sometimes, too, descended to the lauding of quackeries. The report recommended that manuscript books on medical topics, designed for publication, should be referred to the Committee on Medical Literature for critical examination, and that then, if the report was favorable, the work should be produced "under the auspices of the American Medical Association."

In the afternoon work was resumed in the several Sections, and much work of interest and value was accomplished.

In the evening Dr. H. D. Noyes delivered a lecture on "The relation of diseases of the inner structure of the eye to other affections of the body," illustrated by ophthalmoscopic pictures in the magic lantern, in the Medical Department of the University of Pennsylvania, Ninth street, above Chestnut. Prof. R. E. Rogers also delivered a lecture, with demonstrations of electrical phenomena, in the same hall, and receptions of the delegates and their ladies were held by Dr. William H. Pancoast and Dr. Hugh L. Hodge.

---

THIRD DAY.

The Association was called to order at 10 a.m., President Yandell in the chair.

A preamble and resolutions, adopted by the College of Physicians of this city, the preamble reciting the frequency of cases of accidental poisoning by druggists; and the resolutions recommending that all bottles containing poison should not only be labeled poison, but should be roughed on one side so as to indicate their poisonous contents to the sense of touch, and also be labeled with the most ready and efficient antidote, were read, and, upon motion, adopted by the Convention.

A resolution, offered by Dr. Horner, of Virginia, that the members of the Association should discourage the use of alcoholic *stimuli* in their remedies, was adopted.

Dr. Walker, of Brooklyn, presented a resolution that so much of the report of the Committee on Literature as relates to the establishment of a national medical journal, be referred to a committee, to report at the present session. Agreed to.

Dr. Francis Gurney Smith, from the Committee on Nomenclature, read a majority report recommending that the Association adopt a new system of nomenclature, and moved its adoption.

Dr. Woodward, of the U. S. A., read a minority report and accompanying resolution that, as the matter was one too serious for hasty adoption, the majority report be printed and lay over till the next annual meeting.

Some debate ensued, after which Dr. Woodward's resolution was adopted.

Dr. Baldwin read a partial report from the Committee on Nominations, as follows:

President—Dr. Thomas M. Logan, Cal.

First Vice-President—Dr. Catlin, Conn.

Second Vice-President–Dr. McPheeters, Mo.

Third Vice-President—Dr. Pollock, Pa.

Fourth Vice-President—Dr. Briggs, Tenn.

Treasurer—Dr. Caspar Wistar, Pa.

Librarian—Dr. William Lee, D. C.

Committee on Library—Dr. J. M. Toner, D. C.

Next place of meeting—St. Louis.

Assistant Secretary—Dr. M. A. Pallon, Mo.

The report was accepted.

Dr. Baldwin requested further time for his committee to complete their report.

Dr. Logan, of the Committee on Public Hygiene, read a report suggesting that institutions on this branch of medicine be organized throughout the country, so that there might be a more perfect education concerning the matter, and that the medical schools should take more cognizance of the subject, and asking in conclusion that the committee be continued as a Section on State Medicine and Public Hygiene. Report accepted.

OFFICERS OF SECTIONS.

Chemistry and Materia Medica—Dr. R. E. Rogers, chairman, Philadelphia; Dr. Ephraim Cutter, secretary, Boston.

Practice of Medicine and Obstetrics—Dr. D. A. O'Donnell, chairman, Baltimore; Dr. Benjamin F. Dawson, secretary, New York.

Surgery and Anatomy—Dr. Warren, chairman, Baltimore; Dr. F. W. Peck, secretary, Iowa.

Climatology and Epidemics—Dr. George Sutton, chairman, Indiana; Dr. Elisha Harris, secretary, New York.

Medical Jurisprudence, Hygiene, and Physiology—Dr. R. C. Busey, chairman, Washington; Dr. H. B. Arnold, secretary, Baltimore.

Psychology—Dr. Isaac Ray, chairman, Philadelphia; Dr. John Curwin, secretary, Harrisburg.

After the transaction of much other miscellaneous business, the general meeting was adjourned at 1 p.m.

At 3 p. m., work was resumed in the several Sections as on the previous days.

In the evening a lecture on "Sound" was given by Dr. F. Solis Cohen, at the Jefferson Medical College. The doctor illustrated his remarks by the use of various ingenious and delicate apparatus, showing in one case the waves of sound through the intervention of light thrown upon a mirror. The lecture-room was crowded with the delegates and invited guests. After the lecture, a handsome entertainment was given to the members of the Convention and their ladies, by Colonel Thomas A. Scott, at his residence.

FOURTH DAY.

The members of the Association reassembled at 10 a.m., and were called to order by the President, Dr. Yandell.

The session was occupied chiefly in the hearing of the reports from the various Sections; the appointment of the usual number of Standing and Special Committees, as recommended by the Nominating Committee; and the passage of suitable resolutions in relation to several eminent deceased members. After the usual resolutions of thanks, and some appropriate remarks by the President, the Association adjourned for the year. During the afternoon the members enjoyed a very pleasant excursion to the great city park, and an elegant collation provided by the Committee of Arrangements. During the whole four days that the Association held its sessions, the hall of the College of Physicians and Surgeons presented an extensive and interesting collection of surgical instruments and appliances, chemicals, specimens of materia medica, etc., constantly open for the examination of members of the Association. On the whole the meeting was one of the most interesting and successful that has been held; and the Committee of Arrangements are entitled to much credit for the faithful manner in which they discharged their duties.

